# Gastrointestinal perforations by ingested foreign bodies: A preoperative diagnostic flowchart-based experience. A case series report

**DOI:** 10.1016/j.ijscr.2022.107216

**Published:** 2022-05-18

**Authors:** Atef Mejri, Jasser Yaacoubi, Mohamed Ali Mseddi, Ahmed Omry

**Affiliations:** Department of General Surgery, Jendouba Hospital, Tunisia; Faculty of Medicine of Tunis, Tunis El Manar University, Tunis, Tunisia

**Keywords:** Foreign bodies, Gastrointestinal perforation, Peritonitis, Surgery, Laparoscopy, Flowchart

## Abstract

**Background:**

Gastrointestinal tract perforation is the most harmful complication of Foreign Body (FB) ingestion, besides diagnostic delay adversely affects the outcome. This paper aims to present our preoperative diagnostic flowchart and describe the surgical management in a Tunisian center.

**Methods:**

A retrospective review of 48 patients with gastrointestinal perforation by ingested FB treated in the surgery department of Jendouba Hospital. January 2010–December 2020.

**Results:**

48 patients were treated for gastrointestinal tract perforation induced by FB ingestion. The mean age was 56.6 years. The sex ratio was 2/1. Acute abdominal pain was reported in all the patients. 35 patients had abdominal X-ray that showed a FB in 12 cases. CT scan was performed in 38 patients and identified the FB in 28 cases. Postoperative proofreading has identified a preoperative missed diagnosis of FB perforation in 5 cases, all before applying the diagnostic flowchart. All patients underwent open surgery after a median time of 7.12 h. This duration decreased after applying the flowchart (8.21 h versus 5.6 h). 33 patients had a terminal ileum perforation. Enterectomy was performed in 33 patients. Postoperatively, there was one abdominal abscess, one pulmonary embolism, one refractory septic choc, and one wound abscess. The median hospital stay was 6.35 days. The mortality rate was 6.25%. All patients managed with enterostomy had their stoma closed after 3–5 months.

**Conclusions:**

The challenge of gastrointestinal perforation due to FB ingestion is accurate diagnosis and early management. A standardized initial assessment based on a diagnostic flowchart is helpful to achieve this goal and improve outcomes.

## Highlights

• The diagnosis is rarely established preoperatively, as most patients do not recall or disclose ingesting a FB.
• The flowchart makes it possible to suspect the diagnosis in the emergency room.
• Our experience shows that the use of a diagnostic algorithm greatly improves management.
• It is hoped that our experience will encourage other teams to develop diagnostic and even therapeutic flowcharts.

## 1 Introduction

Ingestion of foreign bodies usually occurs in young children or the elderly [1], [2]. Mostly, foreign bodies pass without complications in 80–90% of cases [3], [4], [5], [6]. However, they may lead to severe problems, such as obstruction, bleeding of the gastrointestinal tract, or gastrointestinal perforation, representing a significant health problem with high morbidity and mortality rates [7], [8]. Unfortunately, the diagnosis is rarely established preoperatively, as most patients do not recall or disclose ingesting a FB [2], [5]. CT scan is the modality of choice to confirm the diagnosis and show the lesions' topography [1]. Surgery remains the primary treatment modality, but it is not standardized, and it depends on clinical findings, type, and location of the FB [9], [10], [11]. The outcome depends on early recognition of this condition and prompt management.

We aimed through this paper to present our center's preoperative diagnostic flowchart and describe our management experience.

This case series has been reported in line with the SCARE Criteria 2020 [12].

This case series has been reported in line with the PROCESS 2020 [12].

## 2 Methods

### 2.1 Study design and setting

The study design was based on a descriptive and retrospective analysis. It included patients who underwent surgery for intestinal perforation secondary to ingested FB at the “General surgery department of Jendouba hospital” (located in North-West Tunisia), from January 1, 2010, to December 31, 2020. The “General surgery department of Jendouba hospital” is a tertiary care and teaching department attached to the Faculty of Medicine of Tunis. It is the referral general surgery department of the region serving over 500.000 people. It is a 35 bed-capacity unit. An ethical approval was obtained from the Jendouba Regional Hospital Medical Ethics Committee N° JH58Y21. We confirm that all methods were performed in accordance with the ethical guidelines of the 1975 Declaration of Helsinki.

### 2.2 Study population, data collection, and analysis

All records of patients who were hospitalized for intestinal perforation were reviewed. Only files of patients with a definitive diagnosis of intestinal perforation secondary to ingested FB were included. Data of these patients were obtained from the surgical ward, patient charts, and operation registry books. Exclusion criteria were missing data (information) and patients under 16 years old. Two cases were excluded. Data were collected using a data collection tool including age, gender, ASA, comorbidities, previous abdominal surgery, clinical examination findings, results of biological and radiological examinations, therapeutic procedures used, the emergency status of the procedure, 30-day postoperative complications, 30-day mortality. Statistical analysis was performed using the Statistical Package for the Social Sciences (SPSS) for Windows version 20.

## 3 Results

**Fig. 1:**
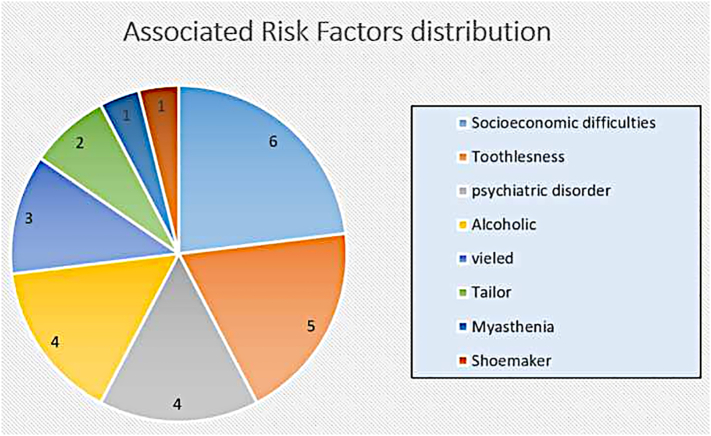
Percentage of associated risk factors in our series.

During the study, 50 patients with gastrointestinal tract perforation by an ingested foreign body were managed at our surgery department. 48 patients were included in this study. The other two were not included because of missing data in their files (data retrieval rate of 94%). The mean age of patients was 56.6 years, ranging from 25 to 72 years. The highest incidence was in the age group of 51–70 years, including 30 patients. There were 32 males and 16 females. In addition, 7 patients had socioeconomic difficulties: one homeless man, one patient who lives in a retirement home, and 5 prisoners. 5 patients had a psychiatric disorder, 1 had myasthenia, 5 patients were alcoholics, 6 patients were toothless, 4 patients were veiled, 3 patients were tailors, and another worked as a shoemaker. Fig. 1 summarizes the Percentage of associated risk factors in our series. According to ASA classification, 13 patients were ASA I, 25 patients ASA II, 10 ASA III. The mean duration of symptoms was 2.16 days and ranged from 1 to 7 days. All patients presented to the emergency department with acute abdominal pain. 32 patients had a fever, 21 had vomiting, 8 had a sub-occlusive syndrome, and 9 patients had generalized abdominal rigidity.

A definitive history of foreign body ingestion was obtained preoperatively in only 9 patients: plastic fragments (prisoner), needles (tailor, veiled), and metal rods (prisoner). Table 1 summarizes the foreign body's nature. Since January 1, 2016, a diagnostic flowchart was elaborated by our Surgery department medical team and approved by our emergency department colleagues, hoping to codify the initial patient approach starting from the emergency room and help avoid diagnostic delay of this devastating condition. The 9 patients who presented generalized abdominal rigidity in the abdominal physical examination were directly taken to the operating room after two-hour resuscitation, including fluid resuscitation, intravenous analgesics and large antibiotherapy, without requesting any radiological examination. 35 patients had a plain abdominal X-ray that showed a FB in 12 cases. An abdominal CT scan was performed in 39 cases. It managed to establish the diagnosis preoperatively in only 7 cases out of 21 (33.3%) before 2016, and was contributive in 16 cases out of 18 (88.8%) after 2016. All patients underwent surgery after a median time of 9.8 h. In our series, 5 patients presented mild abdominal pain and were then hospitalized for uncertain diagnosis and closely monitored. During the hospital stay, they presented abdominal rigidity and had emergency exploratory laparotomy. Surgery delay markedly decreased after applying the flowchart (8.21 h versus 5.6 h).

**Table 1:** Foreign bodies nature.

Foreign body	Frequency	All patients, n = 48
		Percentages (%)
Bones	11	22.91
Toothpicks	8	16.66
Fishbone	5	10.41
Needle	4	8.33
Plastic fragments	4	8.33
Metal rods	2	4.16
Wooden fragment	1	2.08
Vegetable bezoar	1	2.08

Surgeries were conducted by senior surgeons with at least five years surgical specialty experience.

During surgery, 14 had generalized peritonitis, 29 had an intra-abdominal abscess, and one patient had a collection in the hernia sac containing a perforated small bowel loop.

**Fig. 2:**
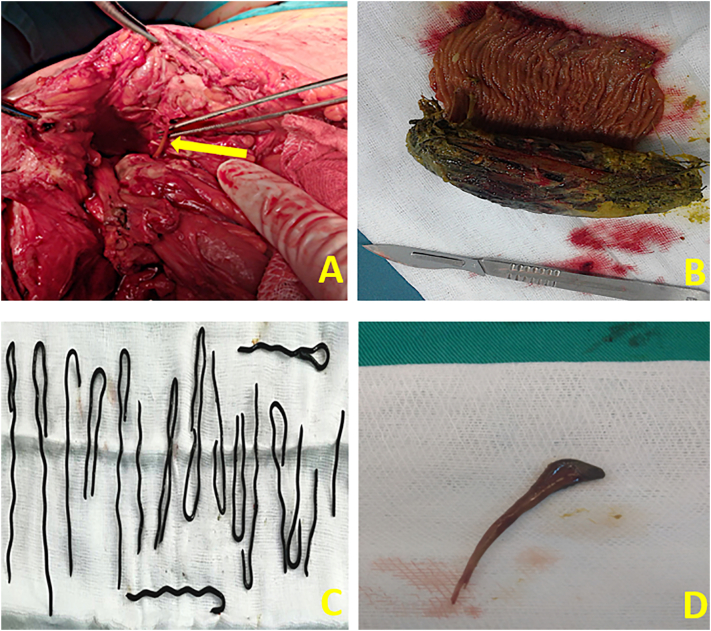
Intra-operative views showing different ingested foreign body: chicken bones (A, D), vegetable bezoar (B), metal rods (C).

**Fig. 3:**
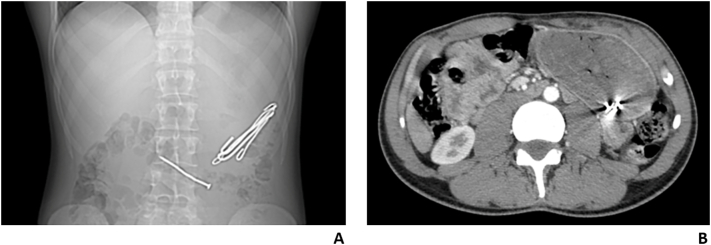
Radiological findings. A: plane radiography B: CT scan.

**Fig. 4:**
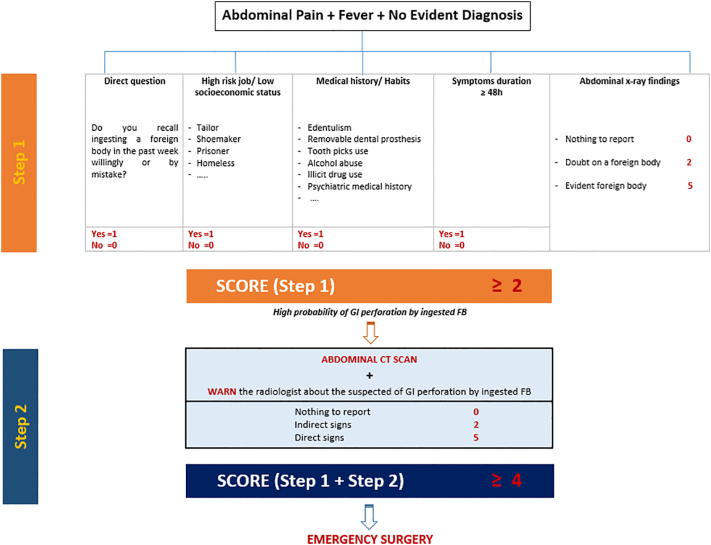
Flowchart.

The most common perforation site was the terminal ileum (*n* = 33, 68.7%) followed by the duodenum (*n* = 6, 12.5%). 4 patients (8.33%) had a perforation in the cecum caused by chicken bones (Fig. 2), one patient had a perforation in the stomach due to metal rods (Fig. 3) and 4 patients had a jejunum perforation caused in one case by a phytobezoar (Fig. 2). The foreign body was found in all patients. The surgical procedure was chosen according to intraoperative features. Enterectomy (small bowel resection) was the procedure of choice in 33 patients (27 underwent an enterostomy and 6 patients underwent an enteroanastomosis), 11 patients had a simple suture, and 4 patients had a right hemicolectomy. Table 2 summarizes the different surgical approaches performed, taking into consideration the peforation location. Postoperatively, one 68-year-old patient with a history of badly followed-up type 1 diabetes had a postoperative abdominal abscess and died during secondary surgery, one patient had a pulmonary embolism on postoperative day 3 and died on postoperative day 7 in the intensive care unit, and one patient died on postoperative day 2 after a refractory sceptic choc. After 2016, only one patient had a postoperative complication. He developed a wound abscess on post postoperative day 4, and he was successfully treated with wound care and intravenous antibiotic therapy. The median hospital stay was 6.35 days, and **it** decreased after using the flowchart (5.5 days vs. 6.96 days). The mortality rate was 6.25%, and all the 3 mortality cases were reported prior to 2016. These findings expressed the value of the initial diagnostic assessment flow chart fixed and relied upon since January 2016. All patients were periodically followed up for a median period of 12 months. All patients managed with enterostomy had their stoma closed after 3–5 months.

**Table 2:** Location and surgical technique.

Location	Technique	All patients, n = 48
		Cases
Stomach	Primary suture	1
Duodenum	Primary suture	6
Jejunum	Primary suture	2
Jejunum	Small bowel resection	2
Ileum	Primary suture	2
Ileum	Small bowel resection	31
Ileocaecal	Right hemicolectomy	4

## 4 Discussion

Ingestion of a FB is not exceptional, and it is usually excreted naturally within week [5]. However, perforation of the gastrointestinal tract is the most feared complication, affecting almost 1% of cases, and when the ingested object is sharp, the perforation rate can be as high as 15%–35% [5], [6]. FB's nature that causes most gastrointestinal perforation is fish bones, chicken bones, and toothpicks.

Patients rarely reveal the ingestion incident, leading to diagnosis delay [13], and patients with palate-reduced sensitivity, toothlessness, psychiatric disorders, or alcohol/drug abuse habits are usually at a higher risk [2]. Furthermore, the ingestion of FB may still occur as part of a suicide attempt or as a work accident [14]. Therefore, direct questions about the incident, the patient's profession, and medical history should figure as part of the history taking in the emergency room.

FB's perforations have been reported in all gastrointestinal tract segments, although it tends to occur in angulation sites of the gastrointestinal tract [6], [15]. The terminal ileum is the most common location of perforation [5]. The manifestation of such perforations can generally be classified into 3 categories: local peritonitis, regional (when it lodges and causes symptoms in an adjacent organ, e.g., liver abscess, pancreatic abscess), or generalized peritonitis [16].

Abdominal pain is the most common symptom. However, symptoms range from mild to life-threatening, and the clinical presentations may mimic diverse surgical emergencies. Overall, symptoms depend mainly on the anatomical lesion [4]. Hence, the diagnosis of a gastrointestinal perforation secondary to the ingestion of a foreign body is not always evident.

The abdominal X-ray in the diagnosis of non-metallic FB's Perforation is usually unreliable [2]. Indeed, in our study, we detected a FB with plain radiography in only 12 out of 35 patients. Furthermore, indirect signs like the pneumoperitoneum are uncommon because the intestinal wall's perforation is usually progressive, allowing the lesion site to be covered by fibrin, omentum, or adjacent small bowel loops [5], [14]. Therefore, the preoperative diagnosis's mainstay is the abdominal CT scan, which identifies the foreign body, location, and lesion's topography with an accuracy ranging between 82% and 90% [1], [17]. Even better, a three-dimensional reconstruction with CT combined with a careful interpretation by an experienced practitioner increases the detection modality's sensitivity. The CT scan diagnosis is based on the direct CT findings, such as discontinuity of the bowel wall and the presence of extraluminal air, and on the indirect CT features, such as bowel wall thickening, abnormal bowel wall enhancement, abscess, and an inflammatory mass adjacent to the bowel [17], [18]. Therefore, a warned radiologist in each case of diagnostic suspicion should search for these valuable signs.

That's why a codified initial assessment (Fig. 4) could improve an early preoperative diagnosis. For example, in our series, before starting to use the diagnostic flowchart in January 2016, more than half (66.6%) of the patients who had CT scans ended up with diagnostic confirmation during surgery, and CT scans diagnostic contribution was obtained after CT scans proofreading, postoperatively, in 5 cases. However, since 2016, the flowchart uses have resorted to a higher level of clinical suspicion. Consequently, it led to better CT scan diagnostic accuracy on preoperative (80% versus 33.3%).

Removing FBs and repairing tissue damage are the two main treatment aims. During the operation, the entire digestive tract must be explored so as not to overlook the concomitant lesions, and surgical management may require trimming the margins and suture, segmentectomy with end-to-end anastomosis, or segmentectomy with a stoma depending on the lesion assessment.

In addition to laparotomy, strategies include laparoscopic, endoscopic, and rarely percutaneous interventional radiological approaches [19], [20], [21]. However, the endoscopic surgery can only be attempted if the FB has not entirely migrated through the gastrointestinal wall and depends a lot on the nature and size of the foreign body, sharp edges, anatomical location, operator experience, and availability of technical equipment, which is expensive [9], [11], [22]. Laparoscopy is currently emerging as the preferred treatment approach for managing complicated FB ingestion [16], [23]. Indeed, it allows a whole peritoneal cavity can be explored adequately with intra or extra-corporeal repair's versatility [16]. Even better, the laparoscopy is relevant, identifying light-reflecting FB and repairing small cross-sectional diameters, such as pins, sewing needles, and fishbone.

## 5 Conclusion

The challenge of gastrointestinal perforation after ingestion of a foreign body is an accurate and early diagnosis. The diagnostic flowchart used in our center was priceless since it offered the ability to improve preoperative diagnostic accuracy. Furthermore, intraoperatively careful exploration is a crucial time not to overlook concomitant lesions. We suggest the diagnostic flowchart as a modality that may help avoid diagnostic errors or delays and improve care coordination among inter-professional team members to decrease morbidity and mortality rates. Additional carefully designed studies are needed further to validate the generalizability of these findings to the larger population.

## Abbreviations

FB: foreign body
CT: computed tomography
ASA: American Society of Anaesthesiologists physical status classification
SPSS: Statistical Package for the Social Sciences

## Consent

Written informed consent was obtained from the patient for publication of this case report and accompanying images. A copy of the written consent is available for review by the editor-in-chief of this journal on request.

## Availability of data and materials

The datasets generated during and/or analyzed during the current study are available from the corresponding author on reasonable request.

## Ethical approval

An ethical approval was obtained from the Jendouba Regional Hospital Medical Ethics Committee N° JH58Y21.

## Funding

None.

## Author contribution

Conceptualization: AM

Data curation: JY, MAM

Supervision and performing surgery: AM

Writing - original draft:AM, AO

Writing - review & editing: AM

The final version of manuscript was read and approved by all authors.

## Guarantor

Atef Mejri.

## Registration of research studies

Not applicable.

## Declaration of competing interest

None.
